# Charting the Chronology of Research on Added Sugars: A Scoping Review and Evidence Map

**DOI:** 10.3390/nu15234974

**Published:** 2023-11-30

**Authors:** Stephen A. Fleming, Jennifer A. Peregoy, Tristen L. Paul, Maria O. Scott, P. Courtney Gaine

**Affiliations:** 1 Traverse Science, 435 E Hawley Street #816, Mundelein, IL 60060, USA; jap455@cornell.edu (J.A.P.); tristen@traversescience.com (T.L.P.); 2 Sugar Association Inc., 1310 L Street, NW, Suite 400, Washington, DC 20005, USA; mscott@sugar.org (M.O.S.); gaine@sugar.org (P.C.G.)

**Keywords:** added sugars, sugar-sweetened beverages, dietary intake

## Abstract

The objective of this study was to conduct a scoping review and produce a publicly available database characterizing the design and reporting elements of the literature on dietary added sugars and select health outcomes. Relevant studies published from 1990 to 2021 were identified to create a database containing information on study and population characteristics, reported added sugars source and concentrations, dietary energy balance, total energy intake, and outcome measures related to body composition, obesity, cardiovascular disease, and diabetes mellitus. There were 245 publications identified, 22% of which describe interventions, and 78% describe observational studies. Publications pertaining to added sugars have risen dramatically since 2010, led by studies primarily assessing body composition (36%) or cardiovascular health (32%), including adults (65%), measuring liquid-only sources of added sugars (56%). Over 65% of studies reported total energy intake, 61% controlled for total energy intake in the design and analysis, and fewer than 5% of studies reported the energy balance of subjects. There has been a significant increase in research on added sugars since 2010, with substantial heterogeneity across all facets of methodology—study designs, exposures and outcomes of interest, terminology, and reporting of dietary intake data—thus limiting the ability to synthesize evidence in this scope of the literature. This evidence map highlights gaps and important areas for improvement to strengthen the state of research and better inform future policies and dietary recommendations on added sugars.

## 1. Introduction

There has been significant debate about sugar intake and health among the global nutrition community. Research on added sugars is particularly challenging due to the complexity of accurately assessing and categorizing added sugars intake. This has yielded a highly heterogeneous body of literature, complicating causal inferences on added sugars intake and health outcomes.

While recommendations to limit the intake of sugars in the diet are not new, the term and specific study of “added sugars” is a relatively recent development. Current authoritative guidance on sugar intake ranges in terms of the definitions of added sugars as well as the rationales and values for intake recommendations. Added sugars are defined by the U.S. Food and Drug Administration (FDA) as sugars that are either added during the processing or preparation of foods (such as sucrose or dextrose), foods packaged as sweeteners (such as table sugar, syrups, and honey), and sugars from concentrated fruit or vegetable juices, excluding sugars naturally occurring in milk, fruits, and vegetables [1]. This definition is similar to that used by the European Food Safety Authority (EFSA) [2]. The World Health Organization (WHO) does not recognize the term added sugars, instead using “free sugars”, which includes added sugars as defined by the FDA as well as fruit juices [3]. Meanwhile, the United Kingdom uses the term “non-milk extrinsic sugars” (NMES), which is defined as sugars not contained within the cellular structure of food, except lactose in milk and dairy products [4]. NMES differs from free sugars in that NMES also account for half of the sugars from dried, stewed, or canned fruit, while free sugars do not consider the processing of fruit.

In recent years, the public health nutrition community has highlighted added sugars as a target for nutritional intervention. Both the 2015 and 2020 Dietary Guidelines for Americans (DGA) recommended that no more than 10% of an individual’s daily calories should come from added sugars [5,6]. This recommendation was based on food pattern modeling, which is designed to help individuals meet nutrient recommendations while staying within calorie needs. It is not based on a threshold associated with adverse health outcomes [5,6]. Similarly, in 2015, the World Health Organization (WHO) strongly recommended a global reduction in the intake of free sugars to less than 10% of total energy intake (TEI) and offered a conditional recommendation to further reduce free sugar intake to less than 5% of total energy intake, based on very low-quality evidence [3]. These recommendations were made on the basis of data linking free sugar intake with the risk of dental caries, not obesity or metabolic diseases.

In 2022, EFSA released a report detailing their efforts to set an evidence-based tolerable upper intake level (UL) for dietary sugars [2]. Following a comprehensive review of the available evidence, EFSA was unable to identify a UL or a safe level of intake for added or free sugars. The agency recommended that intake remain as low as possible. The panel’s inability to define a UL was due to numerous limitations of the data as well as the heterogeneity of the exposures of interest, health endpoints measured, and analytic approaches used. For example, EFSA’s report notes that it was possible to estimate added sugar intake from sugar-sweetened beverages (SSBs) but not foods and that the relationship between dietary sugars and health endpoints is highly dependent on isocaloric comparisons [2].

Several distinct challenges complicate evidence-based reviews of added sugars: the absence of a universally accepted definition for added sugars [7,8], the difficulty of estimating exposures to added sugars [2], the need to consider energy balance [9], and potential differences between food and liquid sources of added sugars regarding their impact on health [10]. To address such challenges, we used evidence mapping to (1) consolidate research used in dietary policy guidance and (2) characterize research on added sugars with a specific focus on food sources (liquids vs. mixed sources of foods and liquids), energy balance, and intake levels. Between sources of added sugars, we specifically investigated what intake levels were most commonly reported, what outcomes were measured, and the extent to which studies controlled for energy balance and reported energy intake. The objective of this review was to produce an evidence map and publicly available database of the body of literature on dietary intake of added sugars and health outcomes. This resource aims to help guide future research efforts and contribute to the development of policy guidance by serving as a resource for relevant research.

## 2. Materials and Methods

### 2.1. Study Selection and Inclusion

The purpose of this evidence map was to capture and characterize studies on dietary added sugars from foods and/or beverages. Both observational and intervention studies assessing outcomes related to body composition, obesity, cardiovascular health, and diabetes mellitus were included.

To assess study eligibility, detailed inclusion and exclusion criteria were developed describing the populations, study designs, exposures, and outcomes eligible for inclusion in the database. Briefly, studies were included if they were primary literature, published in English, were studies on dietary added sugars intake in humans, and measured body weight or composition, obesity, diabetes, or cardiovascular health as an outcome. Detailed inclusion/exclusion criteria are presented in Table 1.

The search strategy employed both a primary literature search and extensive backward citation screening. An electronic search for literature was conducted in PubMed on 12 October 2021 using the following search terms: (“added sugar” [All Fields] OR “total sugar” [All Fields] OR “intrinsic sugar” [All Fields] OR “free sugar” [All Fields] OR “extrinsic sugar” [All Fields]) AND “humans” [MeSH Terms] AND 1990 [EDAT]:2021 [EDAT]. The search was limited to 1990 onwards, as the term “added sugars” is relatively new, and preliminary searches indicated limited in-scope research prior to this period. Search results were further limited to primary literature published in English. No outcome restrictions were set at this stage. A backward citation search was then performed from two authoritative reports that included recommended intakes for added sugars [2,11] to identify additional potentially relevant studies. Specifically, all references cited by the EFSA report and by Chapters 10 and 12 of the DGAC 2020 report were screened. To identify older literature using potentially different terminology, we performed a backward citation search of the relevant chapters of the 2005 Institute of Medicine (IOM) report on dietary reference intakes [12]. Title/abstract and full-text screening was performed using dual-review between 2 and 4 individuals. Conflicts were resolved by a single senior reviewer and through team discussion when necessary.

### 2.2. Data Extraction

A standard operating procedure for data extraction was jointly developed by three reviewers to determine which variables to extract and how those variables should be recorded. Data were not extracted at the comparator level; thus, each included study was only listed once in the database. Three reviewers independently and concurrently performed data extraction in a cloud-based, customized Microsoft Excel spreadsheet, met weekly to discuss the results and continuously updated the data extraction SOP in an iterative, real-time process. All data were dual extracted, with conflict resolution performed by senior reviewers through discussion. The resulting database and description of variables are accessible on GitHub at https://github.com/Traverse-Science/Added-Sugars-Evidence-Map (accessed on 22 October 2023).

To classify data related to exposures to added sugars, the term “SSB” was used to denote the explicit reporting of that category (e.g., a “sugar-sweetened beverage” [13,14,15]), a retail beverage (e.g., a “soda”, “soft drink”, or “fruit drink” [16,17,18]), or an experimentally sweetened solution (e.g., a “glucose-sweetened beverage” [19,20]). Further, where studies reported intake of specific mono- or disaccharides but not total added sugars (e.g., subjects consumed a fructose or glucose drink [21,22]), intakes were classified as “saccharides” and assumed to represent a portion of, but not the total, added sugars consumed in the diet unless otherwise specified. Where studies reported added sugars intake from individual foods or the entire diet, these were extracted as-is (e.g., % energy of added sugars from coffee and tea [23] or %TEI from added sugars [24]). Throughout this report, the term “added sugars” will be used to refer to free sugars, extrinsic sugars, and non-milk extrinsic sugars.

We used a decision-tree approach when extracting added sugars intake data from studies that reported multiple sources or types of added sugars. First, the added sugars exposure that was statistically associated with a health outcome was preferentially chosen. When multiple added sugars exposures were associated with a health outcome, then added sugar intake was preferentially extracted. For example, if total added sugars and soft drink intake were reported, intake of total added sugars was extracted [25]. When intake of added sugars was not available, then the food category (e.g., SSB) was extracted. Otherwise, saccharide intake was extracted (preferentially starting with sucrose). Finally, the intake units preferentially extracted were % TEI, kcal per day, grams per day, and lastly, in serving sizes or volumetric units where available. Where possible, values in grams/day and kcal/day were converted to % TEI using the reported baseline or measured values for TEI. If the intakes of exposures were available at follow-up or the end of an intervention, that value was taken; otherwise, baseline intake was recorded. Finally, intake levels represented a combination of averages (e.g., means or medians [26]), absolute values (e.g., experimentally controlled intake levels [27]), or bins (e.g., quintiles [28]). Sources of added sugars were classified as liquids only vs. mixed (solids and liquids).

Health outcomes were assessed in two ways, both of which were multi-categorical. First, “primary outcome(s)” were extracted according to the primary objective or hypothesis of the study as stated by the authors. Absent a clearly stated primary objective or hypothesis, reviewers assigned the primary outcomes according to the main outcome(s) reported in the study results. These primary outcomes were grouped into 7 categories: body composition (BMI [body mass index], fat mass, body fat percentage, waist circumference, skinfold thickness, etc.), body weight (weight or weight change), obesity (prevalent or incident obesity or overweight), cardiovascular health (lipids, blood pressure, prevalent or incident cardiovascular disease, stroke, etc.), diabetes (prevalent or incident diabetes, glucose metabolism, insulin sensitivity, etc.), metabolism (basal metabolic rate, respiratory exchange rates, metabolic syndrome, metabolic hormones, clinical blood chemistry variables, etc.), and mortality (all-cause mortality and cause-specific mortality).

The second method of extracting health outcomes involved recording all outcomes measured in the study (“measured outcomes”). Studies often measure many variables other than those reflecting the primary outcome(s), making this a more comprehensive and accurate outcome assessment. For example, authors may have described a study’s primary outcome as risk of diabetes as measured by insulin resistance, but also measured liver enzymes [29]. This study would be recorded as having a “primary outcome” in the diabetes category and “measured outcomes” in the glucose metabolism and metabolic measures categories.

Articles were described as containing data on subjects in negative (hypocaloric), positive (hypercaloric), or neutral energy balance only if the energy balance of subjects was explicitly reported. No assumptions based on weight were made to classify the energy balance. Reporting of total energy intake was noted as yes (available) or no (not available). Total energy was marked as being “controlled” (“yes” or “no”) if a study accounted for energy balance in the design (e.g., controlling energy intake of the subjects or maintaining isocaloric balance between comparators) and/or if the statistical analysis included total energy or % TEI from sources of added sugars in the model. For example, a study describing subjects consuming over or at their dietary recommended intake for energy would be labeled as reporting on both “hypercaloric” and “neutral” energy balances, “yes” to reporting of energy if TEI was listed, and “yes” to control of energy if it was included in the statistical model [30].

The data, descriptions of each variable, and methods for extraction are available at https://github.com/Traverse-Science/Added-Sugars-Evidence-Map (accessed on 22 October 2023).

### 2.3. Analysis

Data cleaning, transformation, descriptive analysis, and data visualization were performed in RStudio (Desktop version 2022.7.2.576, http://www.rstudio.com/ (accessed on 22 October 2023)) and Tableau (Tableau Server Version 2022.2.0, https://www.tableau.com/ (accessed on 22 October 2023)) to characterize the included studies by study design, population, exposure, outcomes, and total energy.

## 3. Results

### 3.1. Search Results

**Figure 1:**
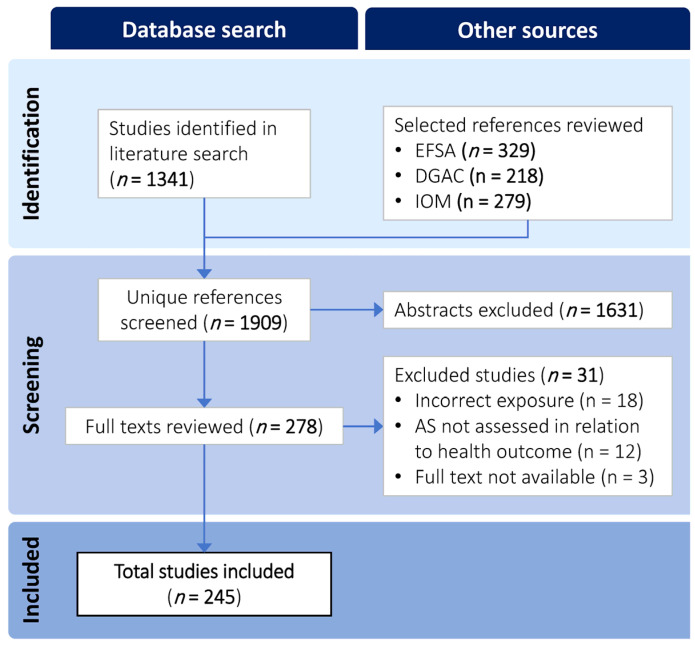
Literature search and selection flow diagram.

The PubMed literature search returned 1341 references, which, combined with the references sourced from the relevant policy documents, yielded 1909 unique references screened at the title/abstract level. A total of 1631 references were excluded after title/abstract screening, leaving 278 full-text articles that were assessed for eligibility. Having met all inclusion criteria, 245 publications were included in the final evidence map and database (Figure 1).

### 3.2. Study Characteristics

**Figure 2:**
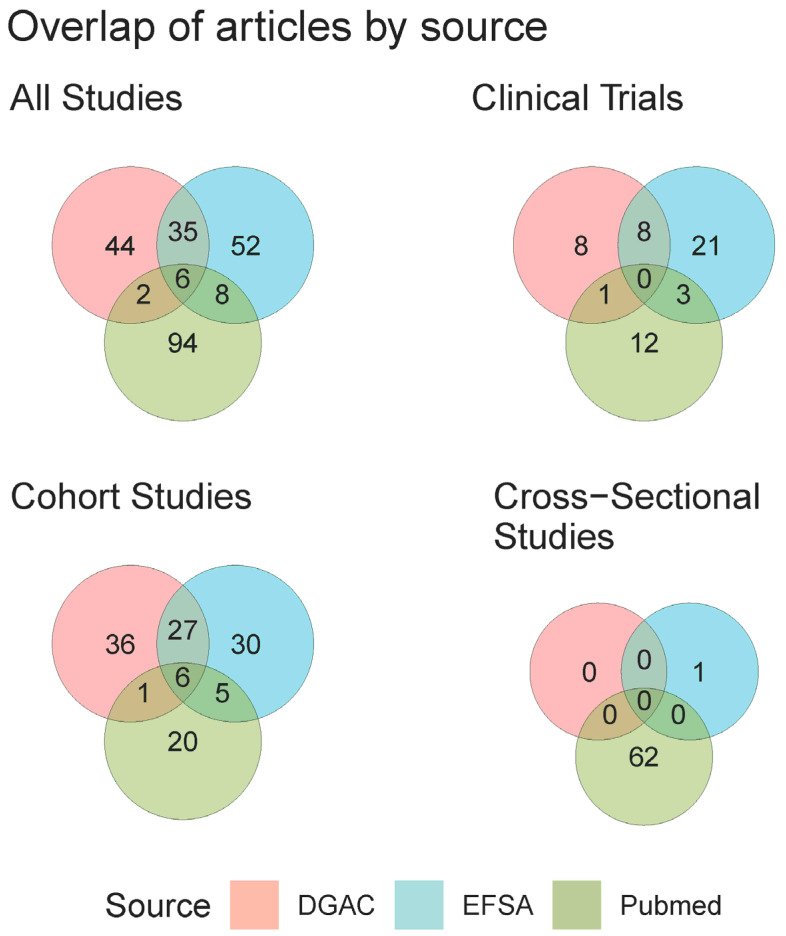
Venn diagram of citation overlaps for all references included in the final database. For visual purposes, “Cohort Studies” includes both case–control and case–cohort studies. References from the IOM were not included in this overlap as they contributed only 5 unique studies.

A total of 245 unique publications met the inclusion criteria. Publications were examined according to source (DGAC 2020, EFSA 2022, and primary literature search) (Figure 2). Only four unique publications were identified from the 2005 IOM report [31,32,33,34]; thus, they are not shown in Figure 2. Six publications (<3% of all articles, all prospective cohorts) were common to all three major sources [15,35,36,37,38,39], whereas 35 publications were cited by both EFSA and DGAC 2020. Overall, there was a low degree of overlap between references from the PubMed literature search and the two reports. The PubMed search returned the only cross-sectional studies, as these were purposefully excluded by the other three authoritative reports and fewer cohort studies. The EFSA and DGAC reports overlapped in 27/125 articles describing cohorts and cited an additional 30–36 unique articles on cohorts each. The EFSA report comprised the most comprehensive source of clinical trials.

Among the 245 included articles, 191 (78%) were observational, and 54 (22%) were interventions (Table 2). Over half (56%) described liquid-only sources of added sugars. Most articles reported on studies of adults (65%, ages 18–64) and a third on children (34%, ages 3–11), with 91% describing participants as healthy or not specifying a specific disease status at baseline. Weight status at baseline included mixed weights for 208 (85%) of the articles, with few exclusively recruiting/analyzing a specific normal, overweight, or obese population. Most studies included multiple primary outcomes, with body composition (36%), cardiovascular health (32%), body weight (22%), and diabetes (22%) being the most frequently reported. Of all included articles, only 13% reported a standardized measure of dietary quality (e.g., DASH score, HEI-2015, etc.); thus, data are not shown here. Of note, there were multiple articles published using data from the same cohorts and surveys. Thus, the number of articles reported in Table 2 is greater than the number of unique interventions, cohorts, and surveys conducted. This information can be viewed in the public database.

### 3.3. Publication Trends

**Figure 3:**
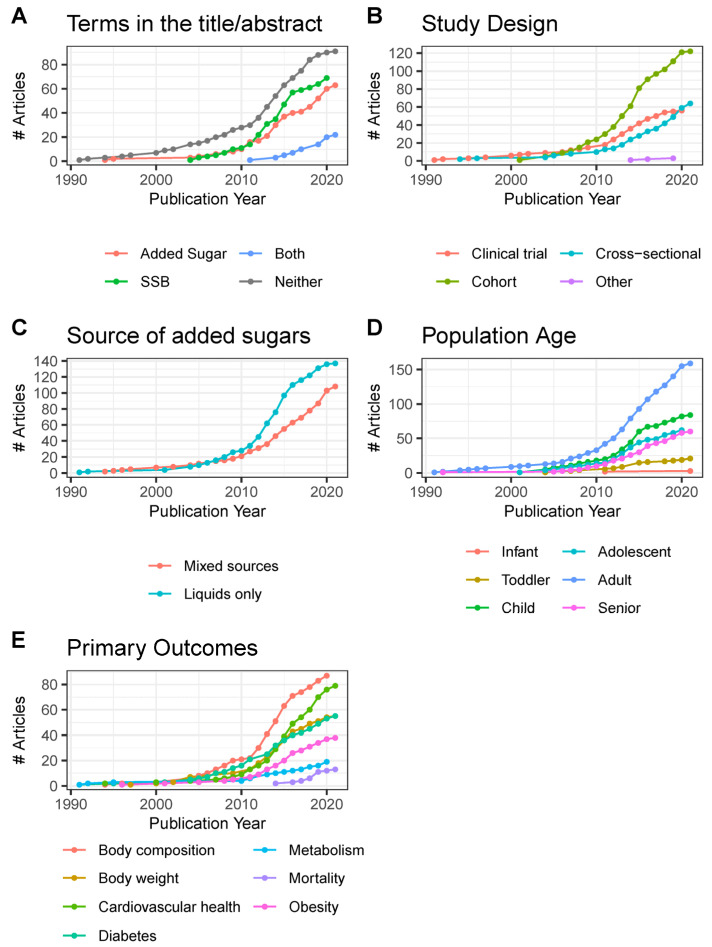
Cumulative growth of publications (*n* = 245) from 1990 to 2021, by (**A**) terms used in the title/abstract, (**B**) study design, (**C**) source of added sugars, (**D**) included age group, and (**E**) primary outcomes. Infant, <12 months old; toddler, 12 months to <3 years old; child, 3–11 years old; adolescent, 12–17 years old; adult, 18–64 years old; senior, 65 years and older. #, Number of.

Published articles on added sugars have increased steadily since about 2010 (Figure 3A). Most articles on the topic of added sugars do not use the phrases “added sugars” or “sugar-sweetened beverage” in titles and abstracts, with few using both (in either their singular or plural forms). The usage of the term “added sugars”, as it relates to the scope of this evidence map, first appeared in 1994. The present evidence map is dominated by cohort study reports, with the number of clinical trials plateauing from 2018 to 2020 and a persistent increase in cross-sectional trials observed starting in 2011 (Figure 3B). Beginning in 2007, articles reporting on studies of added sugars from liquid-only sources eclipsed that of articles on mixed sources (Figure 3C). From 1990 to 2021, the publication of articles examining adult populations has been the most abundant, followed by children and adolescents (Figure 3D).

Starting in 2005, publications reporting on added sugars and body composition as a primary outcome began increasing and then entered a period of steep growth beginning in 2010 that has been sustained through 2020 (Figure 3E). Diabetes was the second most published topic from 2007 until 2014 when articles on cardiovascular health rapidly increased and eclipsed those on diabetes and body weight. Articles specifically assessing obesity as a primary outcome are below articles whose primary outcomes were body weight or composition, but not necessarily obesity. Otherwise, there were few articles that measured outcomes outside of our primary focus, such as general metabolism and mortality.

### 3.4. Intake of Added Sugars

**Figure 4:**
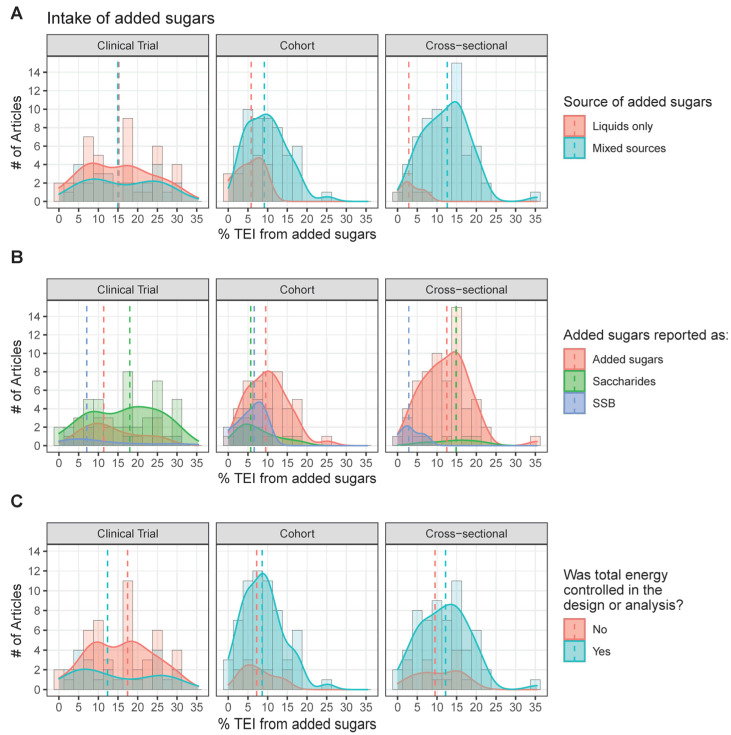
Distribution of reported added sugars intake (% TEI) by study types for (**A**) source, (**B**) form, and (**C**) energy control. Note that this figure represents a subset of the included studies, as only those that quantified added sugars intake at the % TEI level are represented (111/247 studies). These intakes are means, medians, or quartiles for all participants or stratified by participant characteristics (e.g., sex, BMI category, etc.). The dashed line represents the median.

Of all 245 articles, 109 (45%) reported added sugars intake on a % TEI basis. Added sugars were classified as coming from liquid sources (e.g., SSB, experimental sugar solutions, sweetened dairy) or from mixed sources (e.g., granola bars, sweetened yogurt, biscuits, cereal products, bread, jellybeans, SSB and candy, sweet desserts, fruit drinks, and foods not specified). Clinical trials measured intake levels across a wide range from 0 to 30% TEI, regardless of the source. Conversely, cohorts and cross-sectional studies tended to report intake of levels <20%, with intakes of liquid sources at levels <10%, whereas studies reporting intake of mixed sources reported intakes primarily up to 20% TEI (Figure 4A).

Intakes were classified by the source of added sugars according to whether they directly represented added sugars, distinct saccharides (fructose, sucrose, glucose), or SSB. Cohorts and cross-sectionals tended to provide the totality of energy from added sugars (Figure 4B). When added sugars were not reported in observational trials, SSB and then specific saccharides were usually provided. As both SSB and saccharides represent a fraction of the total added sugars, exposures to added sugars from SSB and saccharides appear across a smaller and narrower range than that of total added sugars. The study of specific saccharides was rich across all intake levels in clinical trials, nearly absent in cross-sectional trials, and modestly available from cohort studies.

Intake levels were grouped according to whether or not total energy was controlled for in the study (Figure 4C). There did not seem to be a clear relationship between energy intake from added sugars and total energy being controlled for. Few clinical trials but most cohorts and cross-sectionals controlled for total energy intake. Intake levels were slightly lower, though still similar, in cohort and cross-sectional studies that did not control for energy intake compared to those that did.

### 3.5. Health Outcomes

**Figure 5:**
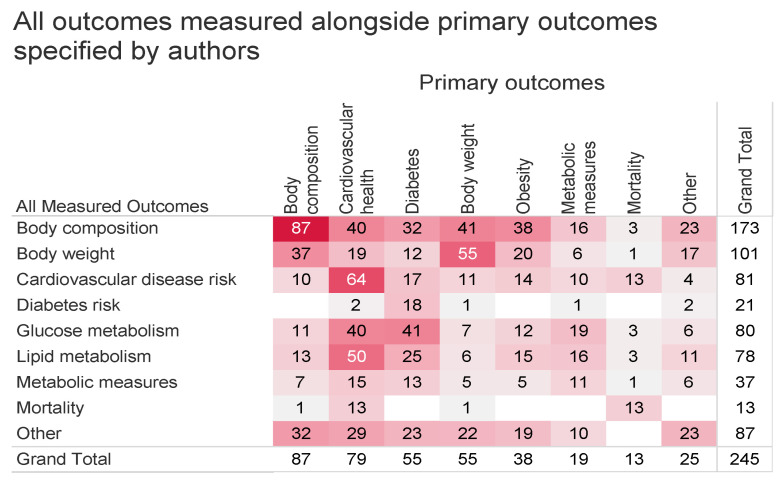
Heat map of all measured outcomes (rows) compared to primary outcomes (columns). “Other” outcomes include risk of metabolic syndrome, inflammatory markers, appetite, dietary intake, and other unrelated outcomes. Color and value indicate the number of articles.

To account for differences in the reporting of primary outcomes and all measured outcome variables, Figure 5 demonstrates the relationship between the two. Across all articles, body composition, cardiovascular health, diabetes, and body weight were the most studied primary and measured outcomes. Overall, there was a high overlap between articles reporting outcomes related to metabolic health, cardiovascular health, and DM. Body composition overlapped with many other outcomes because BMI is a commonly measured outcome. Articles with a primary focus on cardiovascular health frequently measured both glucose and lipid metabolism, whereas articles whose primary focus was diabetes tended to focus more directly on glucose metabolism.

**Figure 6:**
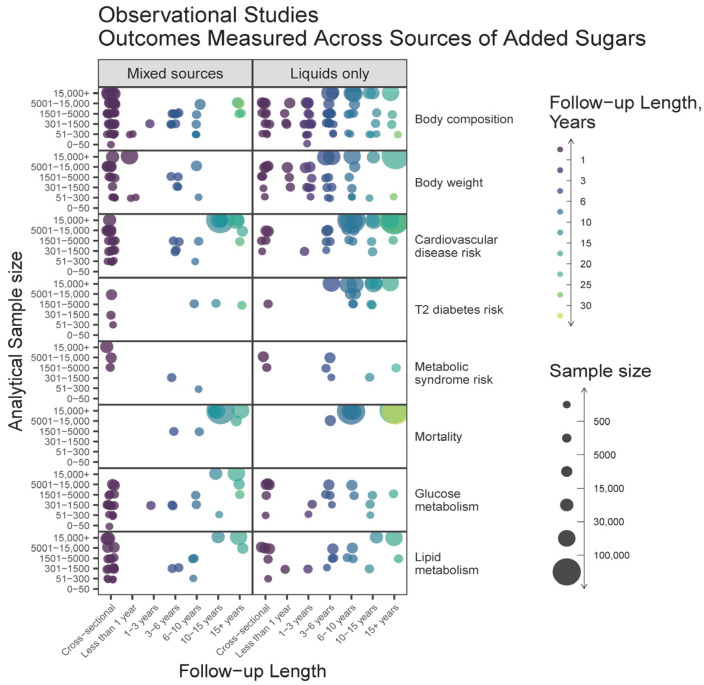
Heat map bubble plot of outcome categories, study duration, and sample size by source of added sugars among observational studies.

**Figure 7:**
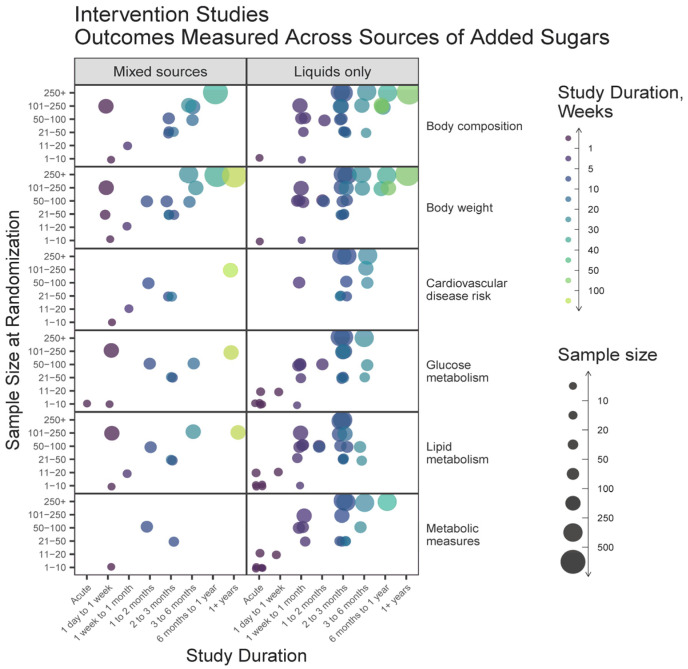
Heat map bubble plot of outcome categories, study duration, and sample size by source of added sugars among intervention studies.

The relationship between measured outcomes, source of added sugars, sample size, and study duration are shown in Figure 6 and Figure 7. Cohorts most frequently followed up between 3 and 15 years, with sample sizes at a wide range between 300 and 15,000 (Figure 6). Overall, the outcomes measured were similar between articles on liquids only or mixed sources. Across all variables measured, articles on liquids only tended to have larger sample sizes and longer durations as compared to articles describing mixed sources. Observational studies with the largest sample sizes and longest durations were those measuring CVD and mortality outcomes.

A high proportion of intervention studies included liquid-only sources of added sugars, and these studies tended to have larger sample sizes and shorter durations compared to studies on mixed sources (Figure 7). For all outcomes, acute studies were conducted more frequently on liquids. The most common intervention durations were less than 3 months. Overall, studies on mixed sources measured similar variables as those on liquid sources but in fewer numbers.

### 3.6. Total Energy Intake and Dietary Energy Balance

Total energy intake was controlled for either in the study design or analysis of 27% of all clinical trials, compared to 76% of cohort studies and 63% of cross-sectional studies (Table 3). Sixty-nine percent of all clinical trials neither controlled for TEI nor specified the dietary energy balance of the participants. Across all study designs, 96% did not specify dietary energy balance. Total energy intake was reported by 59% of clinical trials, 70% of cohort studies, and 69% of cross-sectional studies. Details regarding TEI were also examined by the form of added sugars (liquids only vs. mixed), demonstrating that 29% of mixed-source studies did not report TEI, compared to 36% of the liquids-only studies. The vast majority of studies on liquids and mixed sources did not specify dietary energy balance (98% vs. 93%, respectively).

## 4. Discussion

We used scoping review and evidence-mapping techniques to characterize the literature on added sugars. We found that publications on this topic started to rise significantly in the late 2000s, with peak publication rates between 2010 and 2015. We found that the literature is primarily composed of prospective cohorts and cross-sectional studies, studies reporting intakes of liquid sources of added sugars, studies focused on adult and child populations, and studies measuring body composition and metabolic-related parameters. Like many other assessments [1,2,12], we found significant heterogeneity in study design, subject population, and the exposures reported. Our assessment investigated not only the primary outcomes assessed but all outcome measures collected in each study, in addition to extracting the quantitative intake of added sugars reported and how energy balance was treated in the study design.

### 4.1. Heterogeneity in Terminology Disrupts the Ability to Derive Scientific Conclusions

One of the striking findings from this evidence map was the distinct gap between the literature used for policy guidance and that which fit our inclusion criteria. Although the 2020 DGAC report and EFSA guidance have different scopes and inclusion criteria, both identified over 30 different articles on cohort studies that the other did not include in their analysis (Figure 2). Similarly, EFSA identified 21 clinical trials that were absent from the 2020 DGAC report. Our own literature search identified a completely different set of studies than either document, largely due to our inclusion of cross-sectional studies. The difficulty of performing a systematic review on this topic is highlighted by the fact that many studies that are relevant to the topic use neither the terms “added sugars” nor “sugar-sweetened beverage” (in either their plural, singular, or abbreviated forms) in titles and abstracts (Figure 3A), exacerbating the difficulty of finding applicable literature.

This assessment is not the first to identify numerous challenges to the interpretation of the research due to high heterogeneity. The 2020 DGAC report [11] cites limitations, including a lack of standardization in reporting exposures, such as variations in intake categories, treatment of continuous variables, and the lack of non-linear dose curve assessments. The 2020 DGAC report states that very few RCTs were available for review as the interventions available were ineffective in changing added sugars intake, and many studies were unable to separate behavioral from nutritional effects. Such limitations likely contributed to the DGAC finding insufficient evidence to determine the relationship between added sugars and the risk of CVD.

### 4.2. Research on Added Sugars Disproportionately Emphasizes Liquid Sources

Emerging evidence supports that liquid sources of added sugars may have different impacts on diet quality and health when compared to solid sources [40,41]. For instance, a series of systematic reviews and meta-analyses have demonstrated that the food source alters whether or not fructose intake affects adiposity [42], body weight [43], glycemic control [9], NAFLD [44], and fasting blood uric acid levels [45]. Our results indicate that there is more literature on liquid sources of added sugars (Figure 3C), that observational studies on liquid sources tend to report lower exposures to added sugars compared to studies on mixed sources of added sugars (Figure 4A), and that there are a greater number of large and lengthy studies on added sugars from liquid sources than mixed (Figure 6 and Figure 7). Consequently, available data for policy guidance are biased towards that of liquid sources. This is evidenced by the 2022 EFSA report, which described being able to estimate the intake of sugars from SSBs and 100% fruit juices but not other foods due to the large heterogeneity in reporting [2].

The vast overrepresentation of liquid sources of added sugars in the literature conflicts with real-world exposure to added sugars from various solid sources. The 2020 DGAC report identified that 70% of added sugars intake came from the following five NHANES food categories for ages two and older: sweetened beverages (24%), desserts and sweet snacks (19%), coffee and tea (with their additions) (11%), candy and sugars (9%), and breakfast cereals and bars (7%) [11]. The 2022 EFSA report notes that the food groups contributing the most to added sugars intake are first “sugars and confectionary”, followed by beverages and fine bakery wares [2]. Despite global agreement that food sources of added sugars contribute substantially to added sugars intake, there is a staggering lack of available evidence describing the impact of added sugars from food sources on health outcomes of interest.

We speculate that a major reason for the over-representation of research on added sugars from liquid sources stems from the simplicity of studying SSB. In studies on liquid sources, we found it easier to identify the amount of energy from added sugars because liquids tend to contain fewer or no other nutrients that contribute energy (e.g., soda). Thus, when articles report the amount of energy consumed from soft drinks, the energy can reasonably be assumed to come only from added sugars. This is either more complex or not possible when assessing studies on mixed sources of added sugars. For example, Attuquayefio et al. describe an intervention using a breakfast meal high in saturated fat and added sugars [46]. However, their diet tables only describe “sugar” and do not differentiate between “added sugars” and “total sugars”. While this semantic difference may seem small, the lack of specificity in language inhibits the ability to separate the effects of added sugars from total sugar intake or between liquid and mixed sources.

### 4.3. There Is a Greater Need for Consideration of Energy Intake and Balance

Several reports have emphasized the need to control for energy intake in the study of dietary sugars. For example, a meta-analysis by Choo et al. describes how fructose from sugar-sweetened beverages raises fasting glucose when added on top of the background diet as part of a hypercaloric comparison [9]. Te Morenga et al. also concluded that the “isoenergetic exchange of sugars with other carbohydrates was not associated with weight change” [47]. Indeed, the 2022 EFSA report describes finding no evidence from prospective cohorts (PCs) that the isocaloric exchange of added sugars with other macronutrients is related to any chronic disease they reviewed [2]. However, there is still evidence that an overall positive relationship exists between added and free sugar intake and the risk of obesity and dyslipidemia [2]. A deeper understanding of this relationship is key to making appropriate targets for added sugars intake, reformulation, and achieving improved public health outcomes.

Given that energy balance is a crucial component of the relationship between added sugars and health, it was surprising that clinical trials frequently did not control for energy intake (Figure 3C). While some clinical trials intentionally employed ad libitum designs without statistically controlling for energy intake [21,48,49,50,51,52,53,54], this choice did not always appear deliberate. Beyond controlling for energy intake, 23 of the 56 clinical trials in this evidence map did not report total energy intake at all. In contrast, despite cross-sectional studies suffering from a lack of experimental control, they exhibited much higher rates of measuring added sugars (Figure 3B), reporting TEI, and adjusting for TEI in their statistical models (Table 3). Although 95 of 125 cohorts reported controlling for TEI and 88 of 125 reported the actual TEI, only 1% of cohorts reported the energy balance of their subjects. For example, Jensen et al. reported TEI, estimated total energy expenditure (TEE), and reported the TEI:TEE ratio, demonstrating that children in their study consumed slightly less than they expended [55]. Although they did not use the TEI:TEE ratio in their modeling and noted that the inclusion of TEI in their models did not affect estimates overall, this allowed the authors to better isolate the root differences between groups and measure under/over-reporting intake.

While 150 out of the 245 studies (61%) we assessed controlled for total energy intake (experimentally or statistically), only 12 interventions [19,20,22,31,56,57,58,59,60,61,62,63], one cross-sectional study [64], and two cohorts [30,55] (6% of all studies) explicitly reported the energy balance of their subjects. As most studies collected the data necessary to calculate energy requirements and total energy intake, analyzing, controlling for, and reporting energy balance represents a straightforward and cost-effective approach that most studies could adopt to further the scientific field.

### 4.4. Research Gaps and Opportunities

The results from this evidence map clearly show a bias towards liquid sources of added sugars. Additionally, certain populations are underrepresented in this body of literature—namely, infants, toddlers, seniors, pregnant and lactating women, and obese and diabetic individuals. Fortunately, many limitations are not due to irreversible choices in study design or population recruitment. Rather, improvements in the statistical analysis and reporting of added sugars intake would alleviate numerous concerns. Studies could be strengthened by analyzing and reporting nutrient intakes as an outcome or over time. Energy balance should always be analyzed and reported when the data are available to do so, and when appropriate, both energy balance and nutrient intakes should be included as covariates in multivariate statistical models.

Inconsistencies in terminology significantly impact researchers’ ability to both locate relevant studies on added sugars and draw appropriate inferences from study results. The consistent application of standardized terms for added sugars and SSB would enhance clarity regarding the precise exposure being measured, facilitating the comparison between studies. Improvements can also be made by replacing generic terms like “sugars intake” with more descriptive terms like “total sugars intake” or “total added sugars intake”. Finally, all nutrition science research could benefit from making nutrient intake data publicly available for secondary analysis.

### 4.5. Strengths and Limitations

This evidence-mapping exercise offers numerous strengths. The scraping of references from leading policy documents on added sugars intake ensured that the resultant body of literature included studies deemed relevant by global policymakers. The data extraction process was strengthened by the systematic dual-extraction approach, increasing data reliability. Notably, the comprehensive data extraction approach yielded important information not gathered in previous reviews on added sugars intake, such as the handling of TEI, energy balance, all outcome measures reported, and the intake levels of added sugars.

While this review included numerous systematic elements, it was limited by the inability of database searching to accurately retrieve all related research. The restriction to studies published in English likely introduced geographical and cultural bias in the studies reviewed. More importantly, defining appropriate search terms was challenging as there are no MeSH terms for added sugars. While MeSH terms do exist for sugar-sweetened beverages and dietary sugars, an overwhelming amount of research returned using these terms did not assess added sugars or they described the exposure to added sugars using vague terminology. Thus, we used a combination of phrase searching and backward citation screening to identify relevant research. Backward citation screening was chosen as it is known to be advantageous in reviews where the terminology of interest is inconsistently used [65]. Overall, a stronger literature search strategy would have strengthened this evidence map by capturing a more comprehensive set of relevant publications. However, we note that such limitations due to vague terminology would equally apply to any review of the same topic, as evidenced by the heterogeneity in studies found by the DGAC and EFSA reports (Figure 2). Another notable limitation of this evidence map is that data were not extracted at the comparator level, and therefore, studies reporting multiple sources of added sugars were not fully represented. For example, we prioritized extracting intake data on total added sugars rather than extracting all possible exposures that contributed to added sugars intake (e.g., SSB, sucrose, bakery products, etc.).

## 5. Conclusions

This scoping review and evidence map offer valuable insights into the literature on dietary added sugars and select health outcomes. We observed a significant increase in research on added sugars since 2010, with a notable emphasis on body composition and cardiovascular health in adults, especially concerning exposure to liquid sources of added sugars. There is a broad overlap in the outcomes measured in studies primarily focused on diabetes, cardiovascular health, and body composition. This suggests a substantial body of evidence that provides an opportunity to explore exposure–endpoint associations across related domains. However, our analysis revealed substantial heterogeneity across various methodological aspects, including study designs, exposures, outcomes, terminology, and reporting of dietary intake data. The limited reporting of energy balance and energy intake in these studies raises concerns about potential confounding factors and the comprehensive understanding of the effects of added sugars. Addressing these gaps and improving the quality of research in this field will enhance our knowledge of added sugars’ impact, leading to more informed policies and dietary recommendations for public health. The publicly available database resulting from this research can assist the scientific community in navigating the heterogeneity and identifying relevant studies for future reviews and meta-analyses.

## Figures and Tables

**Table 1:** Study eligibility criteria.

Inclusion Criteria	Exclusion Criteria
Population: Humans of any health status; pregnant women were included if the outcome was measured at the maternal level.	Population: Pregnant women where outcomes were measured at the infant- or dyad-level.
Study Design: All types of intervention and observational study designs (primary literature).	Study Design: Ecological studies; narrative reviews; systematic reviews; meta-analyses.
Exposure: Oral intake of added sugars, free sugars, extrinsic sugars, or SSB. SSBs were defined broadly to include all kinds of sweetened beverages and oral sugar solutions (e.g., sweet tea, lemonade, sports drinks, energy drinks, fruit drinks, sweetened/flavored milks, experimentally created glucose solutions, etc.)	Exposure: Parenteral or enteral nutrition; studies only reporting total or intrinsic sugars; sugar used as an analgesic in infants; label, marketing, or educational studies on consumer perception of sugar; dietary pattern studies where added sugars intake was not directly assessed in relation to a health outcome; whole fruit intake; intervention/exposure groups that did not differ significantly by sugar content/intake but by another nutrient (e.g., fiber); studies assessing the effects of policy/tax changes on added sugars intake.
Outcome: Body weight, body composition, obesity, diabetes, and cardiovascular health; any intermediate biomarkers for these diseases; cause-specific mortality due to these diseases.	Analysis: Studies that did not statistically assess the association between added sugars intake and a prespecified health outcome; studies that only analyzed added sugars intake as a confounder/covariate.
Timeline: Studies from 1990 to 2021.	Language: Non-English publications.

**Table 2:** Summary of study design and population characteristics of studies included in the evidence map and database, stratified by source of added sugars (*n* = 245).

Characteristic	Grand Total	Source of Added Sugars	
		Liquids Only	Mixed
*n* (% of total, column-wise)	245	137	108
Study design			
Parallel arm trial	40 (16%)	30 (22%)	10 (9%)
Crossover trial	14 (6%)	7 (5%)	7 (6%)
Cohort	122 (50%)	85 (62%)	37 (34%)
Cross-sectional	64 (26%)	13 (9%)	51 (47%)
Other	5 (2%)	2 (1%)	3 (1%)
Age group ^1,2^			
Infant	3 (1%)	1 (1%)	2 (2%)
Toddler	21 (9%)	13 (9%)	8 (7%)
Child	84 (34%)	50 (36%)	34 (31%)
Adolescent	62 (25%)	29 (21%)	33 (31%)
Adult	159 (65%)	87 (64%)	72 (67%)
Senior	60 (24%)	29 (21%)	31 (29%)
Baseline health status ^1^			
Healthy	223 (91%)	130 (95%)	93 (86%)
Diabetes	11 (4%)	2 (1%)	8 (8%)
Cardiovascular disease	7 (3%)	4 (3%)	3 (3%)
Other health condition	4 (2%)	1 (1%)	4 (3%)
Baseline weight status			
Exclusively normal weight	9 (4%)	5 (4%)	4 (4%)
Exclusively overweight or obese	28 (11%)	17 (12%)	11 (10%)
Mixed status ^3^	208 (85%)	115 (84%)	93 (86%)
Primary Outcomes ^1,3^			
Body composition	87 (36%)	50 (36%)	37 (34%)
Body weight	55 (22%)	32 (23%)	23 (21%)
Cardiovascular health	79 (32%)	44 (32%)	35 (32%)
Diabetes mellitus	55 (22%)	34 (25%)	21 (19%)
Metabolic measures	19 (8%)	9 (7%)	10 (9%)
Mortality	13 (5%)	6 (4%)	7 (6%)
Obesity	38 (16%)	23 (17%)	15 (14%)
Other	25 (10%)	9 (7%)	16 (15%)

^1^ Not mutually exclusive categories. ^2^ Age categories were defined as follows: infant—<12 months; toddler—12 months to <3 years; child—3–11 years; adolescent—12–17 years; adult—18–64 years; senior—65+ years. ^3^ Primary outcomes represent domains of interest as expressed by the authors but do not represent all measured outcomes.

**Table 3:** Dietary energy balance and total energy intake design characteristics, by study design (*n* = 245).

Characteristic	Clinical Trial (*n* = 56)		Cohort (*n* = 125)		Cross-Sectional (*n* = 64)	
	TEI-Controlled ^1^	TEI Not Controlled For ^1^	TEI-Controlled ^1^	TEI Not Controlled For ^1^	TEI-Controlled ^1^	TEI Not Controlled For ^1^
n (% within column)	15 (27%)	41 (73%)	95 (76%)	30 (24%)	40 (63%)	24 (38%)
Dietary Energy Balance ^2^						
Positive	0	0	1 (1%)	0	0	0
Neutral	6 (40%)	4 (10%)	1 (1%)	0	1 (3%)	0
Negative	2 (13%)	0	1 (1%)	0	0	0
Unspecified	9 (60%)	39 (95%)	93 (98%)	30 (100%)	39 (98%)	24 (100%)
Reported TEI	11 (73%)	22 (54%)	79 (83%)	9 (30%)	32 (80%)	12 (50%)

^1^ Studies were recorded as having controlled for total energy intake if participants were matched on TEI, investigators prescribed a diet with known TEI, or if TEI was statistically adjusted for in the analysis. ^2^ Not mutually exclusive categories.

## Data Availability

The data are accessible on GitHub at https://github.com/Traverse-Science/Added-Sugars-Evidence-Map (accessed on 22 October 2023).

## References

[1] U.S. Food & Drug Administration Added Sugars on the New Nutrition Facts Label. https://www.fda.gov/food/new-nutrition-facts-label/added-sugars-new-nutrition-facts-label.

[2] Turck D., Bohn T., Castenmiller J., de Henauw S., Hirsch-Ernst K., Knutsen H., Maciuk A., Mangelsdorf I., McArdle H., EFSA NDA Panel (EFSA Panel on Nutrition, Novel Foods and Food Allergens) (2022). Scientific Opinion on the Tolerable Upper Intake Level for Dietary Sugars. EFSA J..

[3] World Health Organization (2015). Guideline: Sugars Intake for Adults and Children.

[4] Azaïs-Braesco V., Sluik D., Maillot M., Kok F., Moreno L.A. (2017). A Review of Total & Added Sugar Intakes and Dietary Sources in Europe. Nutr. J..

[5] U.S. Department of Agriculture, U.S. Department of Health and Human Services (2020). Dietary Guidelines for Americans, 2020–2025.

[6] U.S. Department of Health and Human Services, U.S. Department of Agriculture (2015). 2015–2020 Dietary Guidelines for Americans.

[7] Erickson J., Slavin J. (2015). Are Restrictive Guidelines for Added Sugars Science Based?. Nutr. J..

[8] Hess J., Latulippe M.E., Ayoob K., Slavin J. (2012). The Confusing World of Dietary Sugars: Definitions, Intakes, Food Sources and International Dietary Recommendations. Food Funct..

[9] Choo V.L., Viguiliouk E., Mejia S.B., Cozma A.I., Khan T.A., Ha V., Wolever T.M.S., Leiter L.A., Vuksan V., Kendall C.W.C. (2018). Food Sources of Fructose-Containing Sugars and Glycaemic Control: Systematic Review and Meta-Analysis of Controlled Intervention Studies. BMJ.

[10] Yan R.R., Chan C.B., Louie J.C.Y. (2022). Current WHO Recommendation to Reduce Free Sugar Intake from All Sources to below 10% of Daily Energy Intake for Supporting Overall Health Is Not Well Supported by Available Evidence. Am. J. Clin. Nutr..

[11] Dietary Guidelines Advisory Committee (2020). Scientific Report of the 2020 Dietary Guidelines Advisory Committee: Advisory Report to the Secretary of Health and Human Services and the Secretary of Agriculture.

[12] Institute of Medicine (2005). Dietary Reference Intakes for Energy, Carbohydrate, Fiber, Fat, Fatty Acids, Cholesterol, Protein, and Amino Acids.

[13] Ebbeling C.B., Feldman H.A., Osganian S.K., Chomitz V.R., Ellenbogen S.J., Ludwig D.S. (2006). Effects of Decreasing Sugar-Sweetened Beverage Consumption on Body Weight in Adolescents: A Randomized, Controlled Pilot Study. Pediatrics.

[14] Duffey K.J., Gordon-Larsen P., Steffen L.M., Jacobs D.R., Popkin B.M. (2010). Drinking Caloric Beverages Increases the Risk of Adverse Cardiometabolic Outcomes in the Coronary Artery Risk Development in Young Adults (CARDIA) Study. Am. J. Clin. Nutr..

[15] Collin L.J., Judd S., Safford M., Vaccarino V., Welsh J.A. (2019). Association of Sugary Beverage Consumption with Mortality Risk in US Adults. JAMA Netw. Open.

[16] Phillips S.M., Bandini L.G., Naumova E.N., Cyr H., Colclough S., Dietz W.H., Must A. (2004). Energy-Dense Snack Food Intake in Adolescence: Longitudinal Relationship to Weight and Fatness. Obes. Res..

[17] Libuda L., Alexy U., Sichert-Hellert W., Stehle P., Karaolis-Danckert N., Buyken A.E., Kersting M. (2008). Pattern of Beverage Consumption and Long-Term Association with Body-Weight Status in German Adolescents–Results from the DONALD Study. Br. J. Nutr..

[18] Bawadi H., Khataybeh T., Obeidat B., Kerkadi A., Tayyem R., Banks A.D., Subih H. (2019). Sugar-Sweetened Beverages Contribute Significantly to College Students’ Daily Caloric Intake in Jordan: Soft Drinks Are Not the Major Contributor. Nutrients.

[19] Teff K.L., Elliott S.S., Tschöp M., Kieffer T.J., Rader D., Heiman M., Townsend R.R., Keim N.L., D’Alessio D., Havel P.J. (2004). Dietary Fructose Reduces Circulating Insulin and Leptin, Attenuates Postprandial Suppression of Ghrelin, and Increases Triglycerides in Women. J. Clin. Endocrinol. Metab..

[20] Stanhope K.L., Griffen S.C., Bremer A.A., Vink R.G., Schaefer E.J., Nakajima K., Schwarz J.-M., Beysen C., Berglund L., Keim N.L. (2011). Metabolic Responses to Prolonged Consumption of Glucose- and Fructose-Sweetened Beverages Are Not Associated with Postprandial or 24-h Glucose and Insulin Excursions. Am. J. Clin. Nutr..

[21] Aeberli I., Hochuli M., Gerber P.A., Sze L., Murer S.B., Tappy L., Spinas G.A., Berneis K. (2013). Moderate Amounts of Fructose Consumption Impair Insulin Sensitivity in Healthy Young Men. Diabetes Care.

[22] Mark A.B., Poulsen M.W., Andersen S., Andersen J.M., Bak M.J., Ritz C., Holst J.J., Nielsen J., de Courten B., Dragsted L.O. (2014). Consumption of a Diet Low in Advanced Glycation End Products for 4 Weeks Improves Insulin Sensitivity in Overweight Women. Diabetes Care.

[23] Ley S.H., Hanley A.J., Retnakaran R., Sermer M., Zinman B., O’Connor D.L. (2011). Effect of Macronutrient Intake during the Second Trimester on Glucose Metabolism Later in Pregnancy. Am. J. Clin. Nutr..

[24] Halliday T.M., Liu S.V., Moore L.B., Hedrick V.E., Davy B.M. (2018). Adolescents Perceive a Low Added Sugar Adequate Fiber Diet to Be More Satiating and Equally Palatable Compared to a High Added Sugar Low Fiber Diet in a Randomized-Crossover Design Controlled Feeding Pilot Trial. Eat. Behav..

[25] Alderete T.L., Goran M.I. (2020). Added Sugar and Sugar-Sweetened Beverages Are Associated with Increased Postpartum Weight Gain and Soluble Fiber Intake Is Associated with Postpartum Weight Loss in Hispanic Women from Southern California. Am. J. Clin. Nutr..

[26] Herbst A., Diethelm K., Cheng G., Alexy U., Icks A., Buyken A.E. (2011). Direction of Associations between Added Sugar Intake in Early Childhood and Body Mass Index at Age 7 Years May Depend on Intake Levels. J. Nutr..

[27] Umpleby A.M., Shojaee-Moradie F., Fielding B., Li X., Marino A., Alsini N., Isherwood C., Jackson N., Ahmad A., Stolinski M. (2017). Impact of Liver Fat on the Differential Partitioning of Hepatic Triacylglycerol into VLDL Subclasses on High and Low Sugar Diets. Clin. Sci..

[28] Yang Q., Zhang Z., Gregg E.W., Flanders W.D., Merritt R., Hu F.B. (2014). Added Sugar Intake and Cardiovascular Diseases Mortality among US Adults. JAMA Intern. Med..

[29] Goletzke J., Herder C., Joslowski G., Bolzenius K., Remer T., Wudy S.A., Roden M., Rathmann W., Buyken A.E. (2013). Habitually Higher Dietary Glycemic Index During Puberty Is Prospectively Related to Increased Risk Markers of Type 2 Diabetes in Younger Adulthood. Diabetes Care.

[30] Bellissimo M.P., Zhang I., Ivie E.A., Tran P.H., Tangpricha V., Hunt W.R., Stecenko A.A., Ziegler T.R., Alvarez J.A. (2019). Visceral Adipose Tissue Is Associated with Poor Diet Quality and Higher Fasting Glucose in Adults with Cystic Fibrosis. J. Cyst. Fibros..

[31] Ritz P., Krempf M., Cloarec D., Champ M., Charbonnel B. (1991). Comparative Continuous-Indirect-Calorimetry Study of Two Carbohydrates with Different Glycemic Indices. Am. J. Clin. Nutr..

[32] Nuttall F.Q., Gannon M.C., Burmeister L.A., Lane J.T., Pyzdrowski K.L. (1992). The Metabolic Response to Various Doses of Fructose in Type II Diabetic Subjects. Metabolis.

[33] Gibson S.A. (1996). Are Diets High in Non-milk Extrinsic Sugars Conducive to Obesity? An Analysis from the Dietary and Nutritional Survey of British Adults. J. Hum. Nutr. Diet..

[34] Marckmann P., Raben A., Astrup A. (2000). Ad Libitum Intake of Low-Fat Diets Rich in Either Starchy Foods or Sucrose: Effects on Blood Lipids, Factor VII Coagulant Activity, and Fibrinogen. Metabolis.

[35] Lee A.K., Binongo J.N.G., Chowdhury R., Stein A.D., Gazmararian J.A., Vos M.B., Welsh J.A. (2014). Consumption of Less Than 10% of Total Energy From Added Sugars Is Associated With Increasing HDL in Females During Adolescence: A Longitudinal Analysis. J. Am. Heart Assoc. Cardiovasc. Cerebrovasc. Dis..

[36] Sonestedt E., Hellstrand S., Schulz C.-A., Wallström P., Drake I., Ericson U., Gullberg B., Hedblad B., Orho-Melander M. (2015). The Association between Carbohydrate-Rich Foods and Risk of Cardiovascular Disease Is Not Modified by Genetic Susceptibility to Dyslipidemia as Determined by 80 Validated Variants. PLoS ONE.

[37] Warfa K., Drake I., Wallström P., Engström G., Sonestedt E. (2016). Association between Sucrose Intake and Acute Coronary Event Risk and Effect Modification by Lifestyle Factors: Malmö Diet and Cancer Cohort Study. Br. J. Nutr..

[38] Liu Z., Tse L.A., Chan D., Wong C., Wong S.Y.S. (2018). Dietary Sugar Intake Was Associated with Increased Body Fatness but Decreased Cardiovascular Mortality in Chinese Elderly: An 11-Year Prospective Study of Mr and Ms OS of Hong Kong. Int. J. Obes..

[39] Nagata C., Wada K., Yamakawa M., Konishi K., Goto Y., Koda S., Mizuta F., Uji T. (2019). Intake of Starch and Sugars and Total and Cause-Specific Mortality in a Japanese Community: The Takayama Study. Br. J. Nutr..

[40] Sundborn G., Thornley S., Merriman T.R., Lang B., King C., Lanaspa M.A., Johnson R.J. (2019). Are Liquid Sugars Different from Solid Sugar in Their Ability to Cause Metabolic Syndrome?. Obesity.

[41] Wang J., Shang L., Light K., O’Loughlin J., Paradis G., Gray-Donald K. (2015). Associations between Added Sugar (Solid vs. Liquid) Intakes, Diet Quality, and Adiposity Indicators in Canadian Children. Appl. Physiol. Nutr. Metab..

[42] Chiavaroli L., Cheung A., Ayoub-Charette S., Ahmed A., Lee D., Au-Yeung F., Qi X., Back S., McGlynn N., Ha V. (2023). Important Food Sources of Fructose-Containing Sugars and Adiposity: A Systematic Review and Meta-Analysis of Controlled Feeding Trials. Am. J. Clin. Nutr..

[43] Sievenpiper J.L., de Souza R.J., Mirrahimi A., Yu M.E., Carleton A.J., Beyene J., Chiavaroli L., Buono M.D., Jenkins A.L., Leiter L.A. (2012). Effect of Fructose on Body Weight in Controlled Feeding Trials: A Systematic Review and Meta-Analysis. Ann. Intern. Med..

[44] Chiu S., Sievenpiper J.L., de Souza R.J., Cozma A.I., Mirrahimi A., Carleton A.J., Ha V., Buono M.D., Jenkins A.L., Leiter L.A. (2014). Effect of Fructose on Markers of Non-Alcoholic Fatty Liver Disease (NAFLD): A Systematic Review and Meta-Analysis of Controlled Feeding Trials. Eur. J. Clin. Nutr..

[45] Ayoub-Charette S., Liu Q., Khan T.A., Au-Yeung F., Mejia S.B., de Souza R.J., Wolever T.M., Leiter L.A., Kendall C., Sievenpiper J.L. (2019). Important Food Sources of Fructose-Containing Sugars and Incident Gout: A Systematic Review and Meta-Analysis of Prospective Cohort Studies. BMJ Open.

[46] Attuquayefio T., Stevenson R.J., Oaten M.J., Francis H.M. (2017). A Four-Day Western-Style Dietary Intervention Causes Reductions in Hippocampal-Dependent Learning and Memory and Interoceptive Sensitivity. PLoS ONE.

[47] Morenga L.T., Mallard S., Mann J. (2013). Dietary Sugars and Body Weight: Systematic Review and Meta-Analyses of Randomised Controlled Trials and Cohort Studies. BMJ Br. Med. J..

[48] Rasad H., Entezari M.H., Ghadiri E., Mahaki B., Pahlavani N. (2018). The Effect of Honey Consumption Compared with Sucrose on Lipid Profile in Young Healthy Subjects (Randomized Clinical Trial). Clin. Nutr. Espen.

[49] Angelopoulos T.J., Lowndes J., Sinnett S., Rippe J.M. (2016). Fructose Containing Sugars at Normal Levels of Consumption Do Not Effect Adversely Components of the Metabolic Syndrome and Risk Factors for Cardiovascular Disease. Nutrients.

[50] DiMeglio D., Mattes R. (2000). Liquid versus Solid Carbohydrate: Effects on Food Intake and Body Weight. Int. J. Obes..

[51] Yu Z., Lowndes J., Rippe J. (2013). High-Fructose Corn Syrup and Sucrose Have Equivalent Effects on Energy-Regulating Hormones at Normal Human Consumption Levels. Nutr. Res..

[52] Angelopoulos T.J., Lowndes J., Sinnett S., Rippe J.M. (2015). Fructose Containing Sugars Do Not Raise Blood Pressure or Uric Acid at Normal Levels of Human Consumption. J. Clin. Hypertens..

[53] Reid M., Hammersley R., Hill A.J., Skidmore P. (2007). Long-Term Dietary Compensation for Added Sugar: Effects of Supplementary Sucrose Drinks over a 4-Week Period. Br. J. Nutr..

[54] Campos V., Despland C., Brandejsky V., Kreis R., Schneiter P., Chiolero A., Boesch C., Tappy L. (2015). Sugar- and Artificially Sweetened Beverages and Intrahepatic Fat: A Randomized Controlled Trial. Obesity.

[55] Jensen B.W., Nielsen B.M., Husby I., Bugge A., El-Naaman B., Andersen L.B., Trolle E., Heitmann B.L. (2013). Association between Sweet Drink Intake and Adiposity in Danish Children Participating in a Long-term Intervention Study. Pediatr. Obes..

[56] Stanhope K.L., Schwarz J.M., Keim N.L., Griffen S.C., Bremer A.A., Graham J.L., Hatcher B., Cox C.L., Dyachenko A., Zhang W. (2009). Consuming Fructose-Sweetened, Not Glucose-Sweetened, Beverages Increases Visceral Adiposity and Lipids and Decreases Insulin Sensitivity in Overweight/Obese Humans. J. Clin. Investig..

[57] Stanhope K.L., Bremer A.A., Medici V., Nakajima K., Ito Y., Nakano T., Chen G., Fong T.H., Lee V., Menorca R.I. (2011). Consumption of Fructose and High Fructose Corn Syrup Increase Postprandial Triglycerides, LDL-Cholesterol, and Apolipoprotein-B in Young Men and Women. J. Clin. Endocrinol. Metab..

[58] Lowndes J., Sinnett S., Pardo S., Nguyen V.T., Melanson K.J., Yu Z., Lowther B.E., Rippe J.M. (2014). The Effect of Normally Consumed Amounts of Sucrose or High Fructose Corn Syrup on Lipid Profiles, Body Composition and Related Parameters in Overweight/Obese Subjects. Nutrients.

[59] Theytaz F., de Giorgi S., Hodson L., Stefanoni N., Rey V., Schneiter P., Giusti V., Tappy L. (2014). Metabolic Fate of Fructose Ingested with and without Glucose in a Mixed Meal. Nutrients.

[60] Renault K.M., Carlsen E.M., Nørgaard K., Nilas L., Pryds O., Secher N.J., Olsen S.F., Halldorsson T.I. (2015). Intake of Sweets, Snacks and Soft Drinks Predicts Weight Gain in Obese Pregnant Women: Detailed Analysis of the Results of a Randomised Controlled Trial. PLoS ONE.

[61] Stanhope K.L., Medici V., Bremer A.A., Lee V., Lam H.D., Nunez M.V., Chen G.X., Keim N.L., Havel P.J. (2015). A Dose-Response Study of Consuming High-Fructose Corn Syrup–Sweetened Beverages on Lipid/Lipoprotein Risk Factors for Cardiovascular Disease in Young Adults. Am. J. Clin. Nutr..

[62] Vázquez-Durán M., Orea-Tejeda A., Castillo-Martínez L., Cano-García Á., Téllez-Olvera L., Keirns-Davis C. (2016). A Randomized Control Trial for Reduction of Caloric and Non-Caloric Sweetened Beverages in Young Adults: Effects in Weight, Body Composition and Blood Pressure. Nutrición Hosp..

[63] Despland C., Walther B., Kast C., Campos V., Rey V., Stefanoni N., Tappy L. (2017). A Randomized-Controlled Clinical Trial of High Fructose Diets from Either Robinia Honey or Free Fructose and Glucose in Healthy Normal Weight Males. Clin. Nutr. Espen.

[64] Choi A., Ha K., Joung H., Song Y. (2019). Frequency of Consumption of Whole Fruit, Not Fruit Juice, Is Associated with Reduced Prevalence of Obesity in Korean Adults. J. Acad. Nutr. Diet..

[65] Hirt J., Nordhausen T., Appenzeller-Herzog C., Ewald H. (2023). Citation Tracking for Systematic Literature Searching: A Scoping Review. Res. Synth. Methods.

